# Mesenchymal Stem Cells in the Treatment of COVID-19, a Promising Future

**DOI:** 10.3390/cells10102588

**Published:** 2021-09-29

**Authors:** Daniela Gois Beghini, Samuel Iwao Horita, Andrea Henriques-Pons

**Affiliations:** Laboratório de Inovações em Terapias, Ensino e Bioprodutos, Instituto Oswaldo Cruz, Fundação Oswaldo Cruz, Rio de Janeiro 21040-360, Brazil; beghini@ioc.fiocruz.br (D.G.B.); samuelhorita@hotmail.com (S.I.H.)

**Keywords:** mesenchymal stem cells, COVID-19, cell therapy, inflammation, tissue regeneration

## Abstract

Mesenchymal stem cells (MSCs) are multipotent adult stem cells present in virtually all tissues; they have a potent self-renewal capacity and can differentiate into multiple cell types. They also affect the ambient tissue by the paracrine secretion of numerous factors in vivo, including the induction of other stem cells’ differentiation. In vitro, the culture media supernatant is named secretome and contains soluble molecules and extracellular vesicles that retain potent biological function in tissue regeneration. MSCs are considered safe for human treatment; their use does not involve ethical issues, as embryonic stem cells do not require genetic manipulation as induced pluripotent stem cells, and after intravenous injection, they are mainly found in the lugs. Therefore, these cells are currently being tested in various preclinical and clinical trials for several diseases, including COVID-19. Several affected COVID-19 patients develop induced acute respiratory distress syndrome (ARDS) associated with an uncontrolled inflammatory response. This condition causes extensive damage to the lungs and may leave serious post-COVID-19 sequelae. As the disease may cause systemic alterations, such as thromboembolism and compromised renal and cardiac function, the intravenous injection of MSCs may be a therapeutic alternative against multiple pathological manifestations. In this work, we reviewed the literature about MSCs biology, focusing on their function in pulmonary regeneration and their use in COVID-19 treatment.

## 1. Mesenchymal Stem Cells Can Be Isolated from Different Adult Tissues

Mesenchymal stem cells (MSCs) were described by Friedenstein in 1970 [1] and were first isolated from the bone marrow as non-hematopoietic stem cells. In 1991, Caplan introduced the term “mesenchymal stem cells” because it was believed that these cells would differentiate into mesodermal (middle germ layer) cells, such as bone, cartilage, tendon, fat, skin, muscle, and marrow stromal cells [2,3,4]. However, today we know that MSCs can also differentiate into ectodermal [5] and endodermal [6] cell lines, characterizing them as pluripotent stem cells [7,8]. They are adherent spindle-shaped cells found in virtually all adult tissues as a population of undifferentiated tissue-resident cells that facilitate tissue remodeling and repair during life [9]. MSCs generate progeny by self-renewal and can differentiate into various cell types depending on the particular tissue. However, in many organs, their pluripotency decreases with age, leading to reduced regenerative potential.

Although several trials are being conducted using MSCs to regenerate multiple tissues and organs, much remains to be elucidated about their biology and regenerative capacity. In general, MSCs are perivascular cells [10,11] that can be expanded for long periods in culture and, as observed in vivo, extended maintenance in vitro may reduce their pluripotency, limiting their applicability. Moreover, empirical evidence demonstrating that MSCs have the capacity for asymmetric cell division is still lacking [12], a characteristic of conventional stem cells [13]. Additionally, by definition, stem cells are rare cell populations that undergo self-renewal and yield progenitors to differentiate hierarchically into other cell types. However, MSCs appear to affect different cell types and alter the tissue microenvironment via paracrine signaling, inducing differentiation of other resident stem cells (cooperative activity), angiogenesis, chemotaxis, alteration of the local inflammatory response against pathogens and damage [14], tissue repair, proliferation, apoptosis control, and others [15,16].

Finally, MSCs seem to be a much more heterogeneous population than initially envisioned. It is unclear, for example, whether these cells regenerate only in the tissues from which they originate, or if their heterogeneity allows the differentiation into other tissue parenchymal cell types in vivo [3,9]. Moreover, there are no ethical issues involved, in contrast to the use of embryonic stem cells in regenerative medicine, and they can be harvested in relatively large numbers, mainly from bone marrow and adipose tissue [17]. All these characteristics contribute significantly to the therapeutic use of MSCs [14].

**Figure 1:**
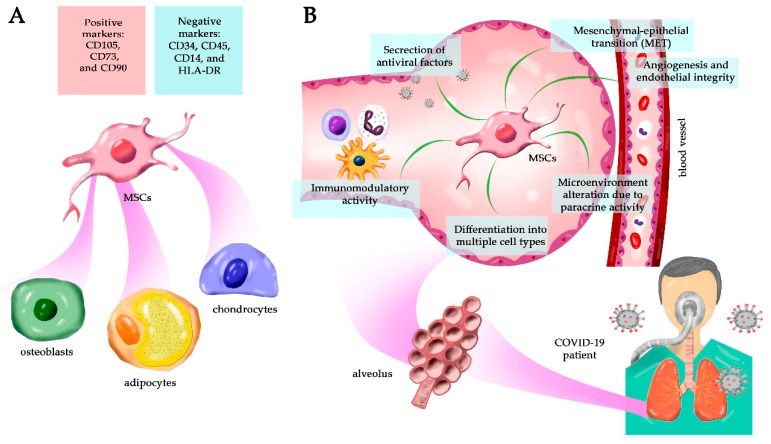
Mesenchymal stem cells (MSCs), general characteristics and function. (**A**) The conventional phenotype for MSCs identification includes CD73, CD90, and CD105 with no CD34, CD45, CD14, and HLA-DR expression. Once isolated, MSCs differentiate into chondrocytes, adipocytes, and osteoblasts in vitro. (**B**) MSCs’ biological functions in treating COVID-19 pneumonia (and systemic disease) include immunomodulatory and antiviral activity, angiogenesis, cell differentiation, mesenchymal–epithelial transition (MET), environment modulation due to the paracrine secretion of soluble factors (secretome), and tissue repair.

Due to the lack of definitive and consensual markers, human MSCs have been minimally identified using their capacity to adhere to plastic culture surfaces, and their canonical phenotype is based on expressed markers after in vitro expansion [18]. The identification of MSCs is usually based on the combined expression of CD73, CD90, and CD105, with no expression of CD34, CD45, CD14, and HLA-DR (Figure 1A). Moreover, it is also necessary that they differentiate into three lineage cells: adipocytes, chondrocytes, and osteocytes, under inductive culture conditions [18]. So far, bone marrow-derived MSCs are the best-characterized cells with multipotent differentiation capacity. These cells have fibroblast-like morphology, adhere to plastic, and undergo extensive proliferation and clonal expansion confirmed by colony-forming unit fibroblast assay (CFU-F). MSCs can also express various additional surface markers, including CD10, CD73, CD140b, CD146, the disialoganglioside GD2, and CD271. Moreover, they may express pluripotency markers such as Oct4, Nanog, and stage-specific embryonic antigen-4 (SSEA-4) that identify different cell populations in the bone marrow [19].

The nomenclature “MSC” causes great confusion in the scientific literature and can also mean “mesenchymal stromal cells”. Thus, the International Society for Cellular Therapy (ISCT) suggests that the fibroblast-like plastic-adherent cells, regardless of the tissue from which they were isolated, be termed “multipotent mesenchymal stromal cells”. Then, the term mesenchymal stem cells should beused only for cells that meet specific stem cell criteria. The widely recognized acronym, MSC, may be used for both cell populations since researchers define the correct designation in their research [20]. In fact, mesenchymal stromal cells are present in the stroma of several organs and tissue and are a heterogeneous population comprising several cell types such as stem cells, progenitor cells, fibroblasts, and many others [21,22]. Thus, it is the stem cells present in this population that can differentiate into cells of the mesodermal, ectodermal, and endodermal lineage, at least in vitro [23]. Therefore, the acronym MSC refers more appropriately to the stem cells defined by the term multipotent mesenchymal stromal cells.

MSCs isolated from different adult tissues are highly heterogeneous. In fact, a comparison of MSCs isolated from bone marrow, umbilical cord blood, or adipose tissue showed diverse characteristics in cell differentiation [24]. With the large number of clinical trials involving MSCs, the scientific community gathered to standardize the studies. This group aimed to prevent the propagation of questionable stem cell-based results and avoid the misuse and commercialization of uncertain stem cell-based treatments. Thus, a consensus was reached with the content of a tool named “DOSES” which is based on the reporting of five core items: D—donor (i.e., autologous, allogeneic, xenogeneic); O—origin of tissue; S—Separation from other cell types/preparation method; E—exhibited cell characteristics associated with behavior; and S—site of delivery [25,26].

## 2. MSCs’ Regenerative Capacity in the Lungs

With the onset of the COVID-19 pandemic and the high mortality rate due to pneumonia and thromboembolic events in the lungs, several initiatives using MSCs started being tested to treat the acute phase of symptomatic patients and pulmonary post-COVID sequelae. The lung is conditionally renewed, and the renovation of epithelial cells in the airways, for example, is less than 1% per day under normal healthy conditions [27]. The lung’s regenerative capacity contrasts with continuously renewed tissues, such as skin and the bone marrow, which, considering proliferation, self-renewal, and differentiation of hematopoietic stem cells (HSCs), generates approximately 1 × 10^9^ hematopoietic cells daily. Similarly to the kidneys and liver, the lungs undergo compensatory growth quickly by increasing the cellular regeneration rate after injury [27].

Several preclinical and clinical trials indicate that MSC-based therapies are safe to treat lung diseases [28]. In 2007, it was demonstrated that plastic-adherent cells could be observed in bronchoalveolar lavage (BAL) fluid from patients that underwent a lung transplant. In this case, clonal proliferation of fibroblast-like cells was observed, and immunophenotypic analysis showed the expression of vimentin and prolyl-4-hydroxylase, suggesting a mesenchymal phenotype. Multiparametric flow cytometry analysis further revealed the expression of CD73, CD90, and CD105, and hematopoietic lineage markers such as CD14, CD34, and CD45 were absent. In addition, they showed multipotency, with the ability to differentiate into adipocytes, chondrocytes, and osteocytes in vitro [29].

MSCs can differentiate into epithelial cells [30], and a remarkable and reversible phenomenon named mesenchymal–epithelial transition (EMT) and epithelial–mesenchymal transition (MET) can occur in embryo development, tumor progression, wound healing, and infections [31] (Figure 1B). In EMT, the natural polarity of epithelial cells is lost due to a profound alteration in the cytoskeleton, associated with the loss of cell–cell adhesion molecules. Moreover, these cells gain migratory and invasive properties and assume an undifferentiated phenotype [32]. This process is triggered by receptors such as the transforming growth factor-β (TGFbRs) and (neurogenic locus notch homolog) NOTCH, and signaling pathways as wingless-related integration site (Wnt) and hypoxia response. Then, transcription factors (TFs) such as ZEB1/2, Snail1/2, Twist, and LEF-1 [33] are activated. These TFs bind and inhibit the expression of genes encoding adherens, tight, GAP junction molecules, desmosomes, E-cadherin, ZO-1, claudins, occludin, and others [34]. While EMT is mainly associated with tumor progression and metastasis, MET leads mostly to wound healing and cicatrization. MET is triggered by growth factor receptors, such as fibroblast growth factor receptors (FGFRs), that bind to FGF10 and -7, for example. This membrane interaction leads to the upregulation of the transcription repressors Sox2 and Oct4 that suppress the EMT mediator Snail1 [35]. Moreover, c-Myc downregulates TGF-b1 and TGF-b receptor 2, and the TF Klf4 activates the epithelial program. These interactions induce epithelial genes such as E-cadherin, EPCAM, MPZL2, STK17A, CLDN3 (claudin), FAM3C, and many others.

Still regarding lung epithelial cells, studies in mice indicate that the lungs contain multiple stem and progenitor cell populations residing in distinct regional niches, besides MSCs [27]. Several types of endogenous airways and alveolar epithelial progenitor cells are distributed in microenvironments throughout the pulmonary tract. Therefore, many pulmonary progenitor cells may also be suitable for future studies and clinical trials, with potential great clinical importance [36,37]. A study used the c-kit (CD117) marker to identify stem cells in the human lung and generated epithelial and mesodermal cell lineages in culture [38]. In another study, human lung stem cells formed functional and structurally integrated bronchioles, alveoli, and pulmonary vessels after injection into damaged mouse lungs in vivo [38]. Another putative lung population of human stem cells with the E-Cadherin^+^ Lgr6^+^ phenotype was isolated and expanded in vitro, showing the ability to self-renew and differentiate in vitro into bronchioalveolar cells. In addition, when the cells were injected into the kidney’s capsule, they produced fully differentiated bronchioalveolar tissue [39]. Using sorted murine EpCAM^+^ epithelial stem/progenitor cells cocultured with lung mesenchymal EpCAM^−^ Sca-1^+^ cells, not MSCs, it was demonstrated that the mesenchymal cells supported the proliferation and differentiation of lung epithelial cells. The authors used a lung injury model and showed the importance of microenvironmental factors, such as FGF10, in epithelial regeneration [40].

Several authors showed that human lung MSCs share common features with bone marrow MSCs. For example, a murine CD45^−^ cell subset also expressing Sca-1, CD73, and CD105 generated a population of cells capable of adipogenic, osteogenic, and chondrogenic differentiation in vitro [41]. Based on that phenotype, the cells were sorted and differed from unsorted lung adherent cells, exhibiting a pattern of gene expression nearly identical to bone marrow-derived MSCs. In BAL fluid, the same authors observed that MSCs from a human asthmatic patient had identical cell surface markers and differentiation potential compared with bone marrow-derived cells [41]. Besides the similarities in phenotype and biological function between MSCs from both compartments, the authors concluded that allergen sensitization and challenge increased the population of lung MSCs. Hoffman et al. also compared adult lung and bone marrow MSCs [42] and showed that MSCs from both compartments are phenotypically and biologically similar in vitro and in vivo after transplantation. However, lung MSCs showed greater survival rates, were more resistant to anchorage-dependent short-term culture, and had enhanced lung retention after the intravenous transplantation. The authors discussed that the increased retention in the lungs was probably due to a higher expression of intercellular adhesion molecule-1 (ICAM-1), platelet-derived growth factor receptor alpha (PDGFRα), and integrin α2 [42]. Moreover, lung MSCs were more efficient in healing elastase-induced injury due to the paracrine anti-inflammatory effect of these cells [42]. Finaly, some studies also showed that MSCs can prevent cell death of alveolar macrophages in vitro by reducing the expression of Bax, a pro-apoptotic protein, and increasing the anti-apoptotic protein Bcl-2 [43].

MSCs also have antimicrobial activity (Figure 1B) and intra-pulmonary cell delivery in a murine model of acute lung injury induced by LPS, improved survival, and mitigated inflammation [44]. MSCs alone have beneficial effects in sepsis induced by cecal ligation and puncture, where they reduced systemic and pulmonary cytokine levels in mice, preventing acute lung injury and organ dysfunction [45]. Using an in vivo mouse model of *E. coli*-induced pneumonia, intratracheal administration of MSCs reduced bacterial growth in lung homogenates and BAL fluid. The BAL itself from MSC-treated mice had a more significant antimicrobial activity than the BAL from PBS-treated mice. This antimicrobial activity was in part mediated by the secretion of the cathelicidin antimicrobial peptide LL-37 [46].

For human treatment, MSCs are generally administered by the intravenous route. Then, it is necessary to understand how these cells home to target tissues [47] since they must interact with endothelial cells and transpose the endothelial barrier to reach damaged or infected tissues. However, while transplanted MSCs flow throughout the recipient’s body, it seems that many of these cells are physically trapped within low-caliber biforked pulmonary vases [48,49]. This possibility may be a partial limitation for MSC-based regimens to treat other damaged tissues; although local vasodilators could easily avoid it, it is a positive characteristic to treat COVID-19 pulmonary impairment.

Regarding the repertoire of adhesion molecules required for cell tethering and adhesion do endothelium, it was demonstrated that MSCs behave much like leukocytes [50]. Then, they require interaction with P-selectin expressed by endothelial cells and the engagement of vascular cell adhesion molecule 1 (VCAM-1) with very late antigen 4 (VLA-4 or CD49d) [51]. The adhesion of circulating MSCs to the endothelium also depends on shear stress due to the flow rate and previous activation of epithelial cells by TNF or IL-1β, but not intercellular cell adhesion molecule (ICAM-1) expression [52]. Although a matter of debate, endogenous MSCs also circulate in peripheral blood at very low frequencies, and their number significantly increases after infection or injury [53]. Moreover, in blood, MSCs may be bound and shielded by platelets and neutrophils, which may affect their capacity to interact with activated endothelium for transmigration [54,55,56].

Understanding all characteristics involved in MSCs biology regarding homing, migration, differentiation capacity, paracrine function, and tissue regeneration will improve the outcome of their clinical use and pave the way for future applications [57], including COVID-19.

## 3. MSCs Are Being Used in Several Treatments

Bone marrow-derived MSCs have been investigated in several in vivo models of lung diseases and are more attractive than embryonic stem cells, as indicated in a previous item [44,58,59,60,61]. They are also more suitable than induced pluripotent stem (iPS) cells, as these cells must be genetically manipulated in a laboratory [62]. MSCs offer great promise for treating several wasting and as yet incurable lung diseases, including emphysema, idiopathic pulmonary fibrosis, pulmonary hypertension, and acute respiratory distress syndrome [63]. One study showed that a population of bone marrow-derived stromal cells transfected with hepatocyte growth factor (HGF) co-expressed MSCs-associated markers, such as CD44, CD29, CD105, CD90, and CXCR4 in rats. To understand whether these cells could have antifibrotic effects, bone marrow MSCs transfected with HGF were injected into fibrotic areas in the lung of rats treated with bleomycin. In this condition, the authors observed that the fibrosis was alleviated [64] (Table 1). The mechanism involved in this beneficial effect has not yet been fully understood, but experimental evidence points to a paracrine activity for repairing injured tissue cells. In a model of bronchopulmonary dysplasia, the intratracheal delivery of bone marrow MSCs on post-natal day four improved survival and exercise tolerance while attenuating alveolar and lung vascular injury and pulmonary hypertension. However, the number of engrafted cells was disproportionately low to solely account for the therapeutic benefit observed, suggesting a paracrine-mediated mechanism [65]. Another study showed the effect of MSCs in a mouse model of ovalbumin (OVA)-induced allergic inflammation and evaluated the effects of systemic administration of human MSCs over allergic inflammation. The authors observed pathology attenuation in many areas, with mucus production and inflammatory cell infiltration inhibition, improved lung function, reduced IgE serum levels, and increased Th2-driven cytokines [66] (Table 1).

In a murine model of Gram-negative-induced pneumonia, the treatment with MSCs enhanced mouse survival and promoted bacterial clearance, which was at least partially due to lipocalin 2 upregulation produced by the MSCs [67]. The application of MSCs was also efficient in controlling influenza infection, leading to reduced lung injury. This effect was attributed to attenuated pro-inflammatory cytokines secretion, reduced recruitment of inflammatory cells, and increased alveolar macrophages [68,69,70] (Table 1). In sulfur mustard-induced lung pathology, one of the most lethal chemicals that leads to major pulmonary complications, the treatment with MSCs was very effective. After intraperitoneal administration of 2-chloroethyl ethyl sulfide (CEES), a sulfur mustard analog, the progression of histopathological changes in the lungs reduced, and the Th1/Th2 balance was restored [71] (Table 1).

Progressive pulmonary inflammation and emphysema have been implicated in chronic obstructive pulmonary disease progression, and the effect of MSCs administration in the pulmonary function was evaluated in rats. The animals were exposed to cigarette smoke for eleven weeks, followed by the administration of MSCs into the lungs. This procedure improved emphysema and destructive pulmonary function by suppressing the inflammatory response, excessive protease expression and apoptosis, and by upregulating vascular endothelial growth factor (VEGF) receptor 2 and TGF-β [72]. The transplant of MSCs into patients with H7N9-induced acute respiratory distress syndrome (ARDS) has already been conducted, and it significantly reduced the mortality of patients in the experimental group, compared with the control group (17.6% against 54.5%) [73] (Table 1).

## 4. MSCs and Their Immunomodulatory Capacity

The primary mechanism of MSC-mediated immunosuppression may vary among different species. For example, immunosuppression of human- or monkey-derived MSCs is mediated by indoleamine 2,3-dioxygenase 1 (IDO1), whereas mouse MSCs utilize nitric oxide (NO) under the same culture conditions [74]. The beneficial effect of MSC-based therapies is multifaceted and may vary according to the target tissues/organs and the nature of the damage. They seem to exert therapeutical activity by enabling the damaged tissues to establish a balanced inflammatory response and regenerative microenvironment. Therefore, MSCs have high plasticity, adopting immunosuppressive or pro-inflammatory phenotypes according to the environment (Figure 1B). For example, it has been published that in the presence of high levels of pro-inflammatory cytokines or after engagement of the Toll-like receptor (TLR)-3 [75], MSCs adopt an immunosuppressive phenotype and reduce cell proliferation and function of multiple cells of the immune system. On the other hand, in the early inflammation phase, with low inflammatory cytokines levels and after TLR-4 engagement, MSCs adopt a pro-inflammatory phenotype and enhance the immune response [75]. These responses are mediated by the release of growth factors, microvesicles, chemokines, cytokines, complement components, immunosuppressive mediators, and many others [76].

**Figure 2:**
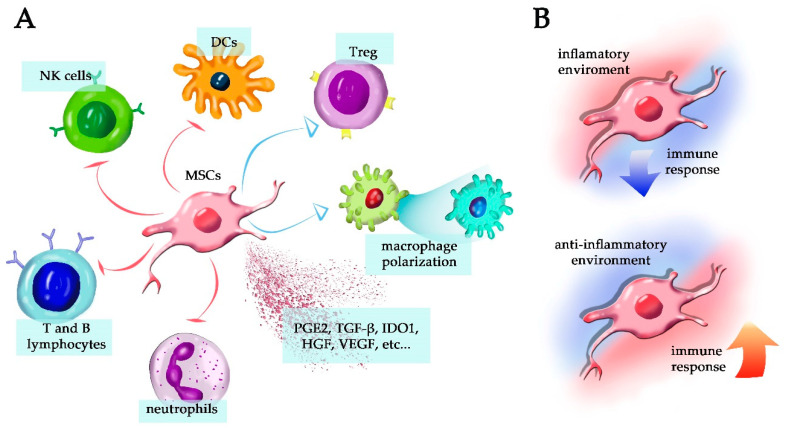
Mesenchymal stem cells and their immunomodulatory capacity. (**A**) In most cases, MSCs are involved in the downregulation of the immune response, silencing cells such as dendritic cells (DCs), T and B lymphocytes, NK cells, and neutrophils. Moreover, they are involved in the induction of cells such as Treg and macrophages polarization towards M2 function. Several factors are also secreted from MSCs, such as prostaglandin E2 (PGE2), TGF-β, hepatocyte growth factor (HGF), vascular endothelial growth factor (VEGF), IDO, and others that can inhibit the inflammatory response. (**B**) The MSCs are dichotomic and can be immunosuppressive when in a pro-inflammatory environment (upper drawing); when in the presence of acute infections or exposed to low levels of pro-inflammatory cytokines, they may potentiate the immune response (lower drawing).

In most cases, however, MSCs are involved in downregulating the immune response by silencing multiple effector T lymphocyte subpopulations and inducing Treg cells (Figure 2A). In addition, they can directly or indirectly inhibit disease-associated Th1-, Th2-, or Th17-mediated cell responses as well as cytotoxic T lymphocytes function, but many central questions remain [77]. In diabetes, it was demonstrated that MSCs that express IL-1Ra, a natural soluble inhibitor of the IL1 receptor, protected the tissue from inflammation-induced injuries and controlled the disease onset [78]. It has also been proposed that MSCs induce T-cell anergy as a potential mechanism of immune suppression, as MSCs lack surface expression of costimulatory molecules, such as CD80 (B7-1), CD86 (B7-2), and others [79].

Through the secretion of multiple inflammatory inhibitors such as prostaglandin E2 (PGE2), membrane molecules such as HLA-G, and cytoplasmic mediators such as IDO1, MSCs inhibit neutrophils, B and T lymphocytes, NK cells, and others (Figure 2A), also attenuate the production of IL-2 [80]. Moreover, MSCs secrete or express IL-10 on the cell membrane [81], produce leukemia inhibitory factor (LIF) [82], galectin-1 and semaphorin 3A [83], and galectin 3 [84], all of which are critical immunosuppressive molecules. Interestingly, interferon-gamma (IFN-γ) and tumor necrosis factor (TNF) are known to enhance the immunosuppressive properties of MSCs, in agreement with the view that these cells downmodulate ongoing inflammatory responses [85] (Figure 2B).

MSCs can also reduce the differentiation of naïve CD4^+^ T lymphocytes into Th1 effector cells or attenuate their function by decreasing IFN-γ production and promoting a shift towards a Th2 immune response. In this case, MSCs downregulated the expression of the natural killer group 2, member D (NKG2D) receptor on CD8^+^ T lymphocytes, then suppressing cell activation [86]. The same study showed that MSCs increased PGE2, IDO1, and TGF-β1 secretion when cocultured with CD8^+^ T lymphocytes [86]. MSCs can also suppress the proliferation of NK cells (Figure 2A). At low NK to MSC ratios, MSCs alter the phenotype of NK cells and suppress cell proliferation, cytokine secretion, and cytotoxicity against HLA-class I-dependent target cells [87]. MSCs have also been shown to inhibit the expansion of blood invariant natural killer T (iNKT) cells and γδT lymphocytes in both cell–cell contact and in transwell systems. This inhibition was mainly mediated by PGE2 [88]. Activated MSCs stimulated by LPS or TNF can also mediate M2 macrophage polarization by releasing PGE2 that acts through the prostaglandin receptors EP2 and EP4, and IL-10 [89].

Melief et al. found that MSCs co-cultured with monocytes induced the secretion of IL-6 and prevented monocyte differentiation into immunogenic antigen-presenting cells [90]. MSCs also skew the differentiation of monocytes towards anti-inflammatory IL-10-producing cells [90]. The authors showed that MSCs promoted monocyte survival and differentiation into CD206^+^ and CD163^+^ type 2 macrophages that secreted high levels of IL-10 and CCL18. Moreover, they observed that MSCs directly induce Treg cells by the secretion of TGF-β and indirectly by triggering the secretion of CCL18 by macrophages that in turn induce more Treg cells [90,91] (Figure 2A).

Among the multiple advantages of using MSCs in cell therapy, their ability to make allosuppression can be included, thus playing a role in immune tolerance and regulation of anti-donor immune responses after solid organ transplant [92]. Apparently, one of the main mechanisms employed by MSCs for allosupression, and immunosuppression in general, is the induction of Treg cells. To date, when MSCs are cocultured with antigen-specific T cells, they induce the expansion of Treg cells [93]. It has also been shown that under inflammatory conditions, MSCs mediate the adhesion of Th17 cells via CCR6 and induce a Treg phenotype in these cells, thereby exerting an anti-inflammatory effect [94]. Moreover, MSCs express Notch 1 ligands (Jagged1, Jagged2, and Delta-Like (DLL) 1, 3, and 4) that activate the Notch-1-dependent signaling pathway in CD4^+^ T lymphocytes, also leading to Treg differentiation [95]. Allogeneic MSCs induced the forkhead box P3 (FoxP3) and CD25 expression (Treg phenotype) by the secretion of prostaglandins and TGF-β and expression of HLA-G5 in coculture with CD4^+^ T lymphocytes [96]. Moreover, when MSCs and B lymphocytes from healthy human donors were cocultured with various B lymphocyte stimuli, cell proliferation and immunoglobulin production were inhibited by the MSCs [97].

In addition to the immunosuppressive activities of MSCs over T and B lymphocytes, they can also inhibit the differentiation and activation of dendritic cells (DCs). This inhibitory effect was shown to be ratio-dependent and resulted in phenotypical and functional modifications in the DCs, as demonstrated by reduced expression of costimulatory molecules and hampered capacity to stimulate naïve T lymphocyte proliferation [98]. MSCs had a profound inhibitory effect on the generation and function of both CD34^+^-derived and monocyte-derived DCs [98]. It was also demonstrated that the coculture of MSCs with DCs led to increased production of IL-10 by the JAK1/STAT3 signaling pathway in DCs, thus inhibiting cell maturation [99]. Collectively, these results indicate that MSCs can modulate immune responses at multiple levels, employing modulatory molecules such as TGF-β, hepatocyte growth factor (HGF), prostaglandins [93,100], IDO1 [101], and others.

Regarding the balance of MSCs as pro- or anti-inflammatory mediators, they may require a ‘licensing’ step provided by inflammatory molecules such as IFN-γ, TNF, or TLR ligands [102]. Probably, the timing of cell exposure to the factors that influence the decision of a pro- or anti-inflammatory role is also critical. Although the inflammatory modulation triggered by TLR engagement remains unclear, the concept of MSCs polarization provides an attractive model to explain some apparently contradictory roles of MSCs in inflammation. Indeed, a pro-inflammatory activity of MSCs may be beneficial in the early phase of inflammation and help mounting a proper immune response [14]. It was published that LPS-stimulated MSCs express chemokine receptors and acquire higher mobility. These stimulated cells secreted large amounts of pro-inflammatory cytokines and recruited neutrophils in an IL-8- and macrophage migration inhibitory factor (MIF)-dependent manner [103]. Although the functional importance of these results remains to be demonstrated in vivo, endogenous MSCs may participate in the early phase of pathogen exposure [103]. Indeed, the balance between apparent opposite biological functions of MSCs depending on the environment further supports their use in therapeutic trials [14], which may, at least in part, also be explained by cellular heterogeneity.

## 5. MSCs Secretome in Tissue Regeneration

As stated before, some main points are considered very important in the biology of MSCs, making them excellent candidates for the treatment of several diseases. These include the fact that sentinel resident MSCs and blood MSCs, which selectively home to injured tissues [51], compose a heterogenous population able to carry out multiple tasks. Moreover, they differentiate into numerous cell types and trigger the differentiation of other local stem cell populations, leading to general tissue repair and angiogenesis. Additionally, they promote tissue inflammation and inflammatory control in the early phase of pathogenic infection. Moreover, their plasticity and adaptation to different tissues and organs make them suitable for systemic injection to treat multi-organ diseases. Many of these functions rely on their capacity to alter the tissue environment through the paracrine secretion of several, and possibly tissue-specific, mediators [104].

The secretome is the supernatant of MSCs maintained in culture under normoxic conditions or in conditioned media and is composed of soluble factors, such as cytokines, chemokines, growth factors, and extracellular vesicles (EV). EVs can be classified into exosomes, microvesicles, and apoptotic bodies depending on their size, biogenesis, and composition [105]. Many of the regenerative properties previously credited to stem cells are being shown to be mediated by secreted exosomes (Figure 1B), and MSCs produce significant amounts of exosomes when compared with other cells [106,107]. The EVs secreted by MSCs are essential, as they preserve most of their parental cell features such as cell homing, immunomodulatory capacity, and regenerative potentials [108]. It is possible, for example, that MSCs-derived EVs act at a distance in vivo, maybe supporting stem cell-based tissue regeneration systemically.

The use of cell-free therapies such as the secretome of MSCs in regenerative medicine provides advantages over cell-based applications, including immune compatibility, tumorigenicity, embolism, and transmission of intracellular infections. Moreover, the storage of MSC-derived EVs can be done without potentially toxic cryopreservative agents and retaining the potency of the sample for an extended period. The MSCs secretome is practical and economical, and samples consisting of either MSCs plus EVs or EVs solely can be obtained from the same cultures. Moreover, the biological composition of secretomes can be modified for more specific and potent effects in therapeutic application.

It was observed that secretome-based therapies improved the pathological condition after cerebral ischemia, myocardial infarction, and trauma [107]. Moreover, it promoted the resolution of LPS-induced lung injury by attenuating lung inflammation and promoting wound healing. It also induced anti-inflammatory M2 macrophages, at least in part by insulin-like growth factor I (IGF-I) activity [109]. The authors also observed that human MSCs secretome reduced the myocardial infarction area in acute patients [110]. Additionally, the secretome contained EVs, HGF, and VEGF, stimulated neurogenesis, and improved traumatic brain injury recovery in a rat model [111]. It was also shown that human adipose-derived MSCs secretome [112] improved tissue reperfusion after ischemia in mice and significantly enhanced endothelial cell growth, bone marrow cells mobilization, and cell homing to the ischemic region [113].

MSCs secretome also contains pro-inflammatory cytokines [114], and the balance between pro- and anti-inflammatory mediators can determine the final effect over the pathogenic condition. Corroborating with this view, some authors showed that MSCs secretome reduced the severity of murine colitis by lowering the pro-inflammatory mediators IL6, IL8, TNF, and MIP-1α transcription, but increasing IL10, an anti-inflammatory cytokine [115]. In another example, the treatment of alkali-injured corneas with MSCs conditioned medium improved wound healing and showed a robust bactericidal effect [116]. The neuroprotective and neurotrophic effects of MSC-derived secretome [110,111] were also demonstrated, with beneficial effects in an animal model of Alzheimer’s disease. This medium contained factors involved in multiple neuroregenerative mechanisms, such as neuroprotection, axonal elongation, neurotransmission, inflammation suppression, and microglial regulation. Notably, secretomes also attenuated pro-inflammatory responses induced by β-amyloid plaques and generated an anti-inflammatory/tissue regeneration environment, accompanied by the induction of anti-inflammatory M2-like microglia [117].

The role of MSCs in angiogenesis is of great interest due to the large number of diseases related to insufficient or abnormal vessel growth, such as arteriosclerotic diseases. In this regard, some studies have shown that MSCs secrete factors that induce proliferation and migration of endothelial cells, promoting tube formation and preventing endothelial cell apoptosis in vitro [118]. Moreover, the secretion of pro- and anti-angiogenic factors can be modified by hypoxia conditions [119].

Purified exosomes from human MSCs culture reduced the infarcted area in a mouse model of myocardial ischemia/reperfusion injury [120]. Moreover, microvesicles derived from human adult MSCs protected from acute kidney injury induced by ischemia-reperfusion and subsequent chronic renal damage [121]. MSC-derived microvesicles also ameliorated lung inflammation, and their therapeutic effects and immunomodulatory properties were partly mediated by angiopoietin-1 [122]. In another study, bone marrow-derived MSCs microvesicles played a protective role in acute pancreatitis by reducing the levels of pro-inflammatory cytokines and regulating the nuclear translocation of NF-κB p65 [123]. MSC-derived exosomes promoted liver regeneration in drug-induced liver injury models [124], and exosomes derived from MSCs also exerted a cardioprotective effect after myocardial ischemia/reperfusion injury through the activation of the Wnt/β-catenin signaling pathway [125].

MSCs have also been suggested for the treatment of tumors, although some contradictory results were obtained. It was observed that MSC-derived microvesicles inhibited the tumor bladder T24 cells growth in vitro and in vivo [126]. The in vivo intra-tumor administration of MSCs microvesicles in already established tumors in SCID mice significantly inhibited tumor growth [127]. An additional advantage of using exosomes to treat tumors is that they can be engineered to contain relevant gene information and retain the ability to home to the tumor site and be internalized by the target cells [107,128].

Recently, exosomes derived from MSCs were proposed as a possible therapy to treat the “cytokine storm” observed in COVID-19 patients with severe pneumonia [129], but a limited number of human clinical studies using MSCs is available. Furthermore, it was evaluated if MSCs transplantation would improve the outcome of seven enrolled patients with COVID-19. The pulmonary function and symptoms of those patients were significantly improved within two days after MSCs transplantation. After three to six days of treatment, the overactivated inflammatory CXCR3^+^CD4^+^ T cells, CXCR3^+^CD8^+^ T cells, and CXCR3^+^ NK cells were not observed. In addition, a group of CD14^+^CD11c^+^CD11b^mid^ regulatory DC population dramatically increased, and the intravenous transplantation was proven safe and effective, especially for patients critically ill [130]. Currently, ClinicalTrials.gov and the WHO Clinical Trials Registry Platform (WHO ICTRP) report a combined 28 trials exploring the potential of MSCs or their products for COVID-19 treatment [131].

## 6. MSC As an Alternative Treatment for COVID-19

The COVID-19 pandemic is caused by the virus SARS-CoV-2, which spread quickly and became a global health emergency. The rapid virus replication in the lungs triggers a strong inflammatory response that leads to a “cytokine storm”, causing ARDS and respiratory failure, the leading cause of death in severely affected patients [132]. Although the lungs are more evidently compromised, the disease affects multiple organs, and patients with a previous history of cardiac symptoms, obesity, diabetes, or coagulation disorders, for example, have a higher morbimortality risk. Then, considering COVID-19 a systemic disease, we can assume that multiple affected organs and tissues could benefit from the treatment with MSCs and/or with their secretome.

In the lungs, the inflammation is caused by the release of cytokines virally triggered, including IL-2, IL-6, IL-7, granulocyte colony-stimulating factor (GSCF), interferon-gamma-induced protein 10 (IP10), monocyte chemoattractant protein 1 (MCP1), macrophage inflammatory protein 1α (MIP1α), and TNF. These mediators and others lead to pulmonary edema, air exchange dysfunction, and acute cardiac injury, for example [133]. Currently, available therapies include non-specific antivirals, antibiotics to prevent secondary infection and sepsis, and corticosteroids to reduce inflammation. However, the success rate of this therapeutic strategy is low in patients with severely affected pulmonary function. Therefore, several drugs are being tested for repositioning, including hydroxychloroquine, Remdesivir, and Ivermectin worldwide [134]. Among cell-based alternatives, MSCs have more registered clinical trials and possibly more chances to be approved for COVID-19 treatment [135]. Moreover, they have documented safety profiles from FDA-approved clinical trials. A systematic review of several clinical trials involving over one thousand participants that received intravascular transplants of MSCs for different diseases and clinical conditions was performed in 2010. In that study, a follow-up was done varying from two weeks to thirty months and attested to the safety of MSCs therapy [136].

A clinical study of severely affected COVID-19 patients using MSCs derived from the human umbilical cord was performed. The patients were divided into the standard treatment group and the standard treatment group that received the cell infusion. After twenty-eight days, the mortality in the group that received cell infusion was 0% and in the other group was 10.34%. Moreover, the clinical condition improved from the third day after cell infusion and reached a significant difference on the seventh day. There was also a significant reduction in C-reactive protein and the level of IL-6, and the number of lymphocytes returned to the normal range significantly faster [137]. A study using MSCs from adipose tissue was also carried out in thirteen patients with COVID-19 on mechanical ventilation. There was a clinical improvement in 70% of the patients, on average sixteen days after the first application. Seven patients were extubated, and four remained intubated (two with an improvement in their ventilatory and radiological parameters and two in stable condition). Two patients died, but particularly in those patients with clinical improvement, there was a decrease in the inflammatory and coagulation parameters, C-reactive protein, IL-6, ferritin, LDH, and d-dimer, as well as an increase in lymphocytes [138]. Another study evaluated the allogeneic transplantation of menstrual blood-derived MSCs in two hospitalized patients. The first patient received MSCs transplantation through intravenous infusion three times on February 5th, 6th, and 8th of 2020. The injection dose of MSCs was set to one million cells per kg of body weight. The second patient received the same intravenous infusion three times on February 8th, 9th, and 11th of 2020. Then, the fraction of inspired O_2_ of the two patients gradually decreased while the oxygen saturation and partial oxygen pressure improved. Furthermore, the patients’ computed tomography (CT) scans showed that bilateral lung exudate lesions were adsorbed after MSCs infusion [139].

In a case study, a 65 year-old patient was diagnosed with COVID-19, and after twelve days of treatment, the patient’s symptoms and inflammation were still severe. Then, allogenic human umbilical cord MSCs were given three times (5 × 10^7^ cells each time) with a three day interval, with thymosin α1 and antibiotics daily. After treatment, most laboratory indexes and CT images showed inflammation remission [140].

In another study, twenty-five patients with severe COVID-19 received a clinical grade, and MSCs were given at a dose of 1 × 10^6^ cells per Kg. Promethazine hydrochloride (intramuscular injection, 25 mg) was used before the injection of MSCs to prevent allergies. Seven individuals received MSCs once, other seven patients received MSCs twice, and eleven individuals were infused with cells three times. After MSCs therapy, sixteen cases (64%) showed CT scan improvement, and all cases gained clinical improvement. No fatalities occurred during hospitalization, but none of the inflammation indexes changed significantly after MSCs therapy. In addition, the serum levels of lactate (LAC), cardiac troponin T (cTnT), and creatine kinase MB (CK-MB) elevated significantly after MSC therapy. The authors discussed that maybe no immunomodulation was observed because the optimal detection time frame was lost. Finally, MSCs therapy appears to be a promising option for treating severe COVID-19 but should be used with caution, especially in patients with coronary heart disease and metabolic acidosis [141].

As discussed, the importance and immunomodulatory effects of MSC-derived EV in vitro emerged as a potential tool for treating COVID-19. This is due to a marked anti-inflammatory effect and regenerative capacity in various in vitro and in vivo models of the disease [142,143]. MSC-derived EVs are also promising in treating post-COVID-19 pulmonary fibrosis, described as a complication in COVID-19 patients [144]. This possibility is reinforced by the observation that MSC-derived EVs can prevent the development of fibrosis following experimental lung injury [145].

In one of the studies using MSCs to treat COVID-19, exosomes derived from allogeneic bone marrow MSCs (ExoFlo) were used in twenty-four SARS-CoV-2 PCR-positive patients. Patients with moderate-to-severe ARDS received a single 15 mL intravenous dose of ExoFlo and were evaluated for treatment safety and efficacy for fourteen days. The results were very encouraging, with no adverse effects, and reached an 83% survival rate. Of the total patients, 71% recovered, 13% remained critically ill though stable, and 16% died for reasons unrelated to the treatment. Laboratory results revealed a reduction in blood neutrophils, CD4^+^, and CD8^+^ lymphocyte numbers in recovering patients. Likewise, acute phase reactants declined, with mean C-reactive protein, ferritin, and d-dimer reduction [146].

## 7. Conclusions

MSCs have several advantages over other populations of stem cells, including their maintenance in vitro for long-term clonal expansion, generation of secretome, and the possibility for in vitro manipulation to improve cell properties. Moreover, they can migrate and disperse in different tissues, adapting to the biological demands and necessities locally with shallow risk after in vivo injection. Then, they may populate different organs and tissues and affect the environment by the paracrine secretin of local mediators and be affected by the environment, acquiring specific functions. Much remains to be understood about MSCs biology, but they pose a promising tool for treating systemic diseases such as COVID-19.

## Figures and Tables

**Table 1:** Different pulmonary diseases and the described effects of MSCs treatment.

Disease and Disease Model	MSCs Origin	Pathogeny Improvement	References
Bleomycin-induced usual interstitial pneumonia	Rat stromal cells	Reduced fibrosis	[64]
Bronchopulmonary dysplasia	Rat	Increased mice survival and improved exercise tolerance	[65]
OVA-induced allergy	Human MSCs intravenously injected into mice	Reduce mucus, inflammation, improved pulmonary function	[66]
Bacteria-induced pneumonia	Mouse	Increased mice suvival and bacterial clearance	[67]
Incluenza infection	Mouse	Reduced lung injury	[68,69,70]
Sulfur mustard-induced lung injuty	Mouse	Improved pulmonary function, Altered Th1/Th2 balance	[71]
emphysema	Rat	Improved pulmonary function	[72]
Acute respiratory distress syndrome (ARDS)	Human	Reduced mortality	[73]

## Data Availability

Not applicable.
